# Primary care physicians are under-testing for celiac disease in patients with iron deficiency anemia: Results of a national survey

**DOI:** 10.1371/journal.pone.0184754

**Published:** 2017-09-20

**Authors:** Marisa Spencer, Adrienne Lenhart, Jason Baker, Joseph Dickens, Arlene Weissman, Andrew J. Read, Seema Saini, Sameer D. Saini

**Affiliations:** 1 Department of Internal Medicine, University of Michigan, Ann Arbor, Michigan, United States of America; 2 Department of Internal Medicine, Henry Ford Health System, Detroit, Michigan, United States of America; 3 Department of Statistics, University of Michigan, Ann Arbor, Michigan, United States of America; 4 Research Center, The American College of Physicians, Philadelphia, Pennsylvania, United States of America; 5 Veterans Affairs Center for Clinical Management Research, Ann Arbor, Michigan, United States of America; 6 Ambulatory Care, Veterans Affairs Medical Center, Ann Arbor, Michigan, United States of America; Pennsylvania State University College of Medicine, UNITED STATES

## Abstract

**Background:**

Iron deficiency anemia (IDA) is a common extra-intestinal manifestation of celiac disease (CD). Little is known about the frequency with which primary care physicians (PCPs) test for CD in patients with IDA. We aimed to describe how PCPs approach testing for CD in asymptomatic patients with IDA.

**Methods:**

We electronically distributed a survey to PCPs who are members of the American College of Physicians. Respondents were asked whether they would test for CD (serologic testing, refer for esophagogastroduodenoscopy [EGD], or refer to GI) in hypothetical patients with new IDA, including: (1) a young Caucasian man, (2) a premenopausal Caucasian woman, (3) an elderly Caucasian man, and (4) a young African American man. These scenarios were chosen to assess for differences in testing for CD based on age, gender, and race. Multivariable logistic regression was used to identify independent predictors of testing.

**Results:**

Testing for CD varied significantly according to patient characteristics, with young Caucasian men being the most frequently tested (61% of respondents reporting they would perform serologic testing in this subgroup (p<0.001)). Contrary to guideline recommendations, 80% of respondents reported they would definitely or probably start a patient with positive serologies for CD on a gluten free diet prior to confirmatory upper endoscopy.

**Conclusions:**

PCPs are under-testing for CD in patients with IDA, regardless of age, gender, race, or post-menopausal status. The majority of PCPs surveyed reported they do not strictly adhere to established guidelines regarding a confirmatory duodenal biopsy in a patient with positive serology for CD.

## Introduction

Celiac disease (CD) is an immune-mediated disorder triggered by exposure to dietary gluten in genetically susceptible individuals. Previously, CD had been described predominantly as a pediatric disorder; however, it is now increasingly recognized in adult patients, including the elderly [1]. CD currently affects around 1% of the general population [2–6], and the prevalence of CD appears to be increasing over time [1]. Although CD is thought to primarily affect non-Hispanic Caucasians, other ethnicities can also develop CD, though the data on prevalence is less robust [7,8].

Despite an overall increase in awareness, CD is still an under-diagnosed condition [9]. Indeed, the diagnosis is often delayed by years, which may reflect the non-specific symptoms of CD and a low index of suspicion by providers. Patients can present with subtle, extra-intestinal manifestations of disease, such as iron deficiency anemia (IDA) or osteoporosis. IDA represents the most common extra-intestinal manifestation and has been reported to occur in up to 50% of patients diagnosed with subclinical disease [5,10–12]. Conversely, CD has been recognized as the underlying etiology of unexplained IDA in up to 7% of encountered cases [10,11,13–16]. As a result, clinical practice guidelines from groups such as the American Academy of Family Physicians (AAFP) and the British Society of Gastroenterology (BSG) suggest testing for CD in patients with unexplained IDA. Yet, we know little about how physicians currently approach work-up in such patients [17,18].

The purpose of this study was to describe how primary care physicians (PCPs) approach the work-up of unexplained IDA in asymptomatic and minimally symptomatic patients, and more specifically, to determine the frequency of testing for CD in this population of patients. In addition, we sought to identify which patient- and physician-level factors predicted the use of testing for CD.

## Methods

### Overview

We developed a 25-item multiple-choice survey to assess how PCPs work-up unexplained IDA. The primary outcome was frequency of testing for CD with: (1) serologic testing; or, (2) any testing (which included serologic testing, “open access” EGD, or referral to gastroenterology). Additionally, because initiation of a gluten free diet (GFD) will reduce the sensitivity of duodenal biopsies for disease confirmation, we also sought to quantify the proportion of respondents who would confirm the diagnosis of CD with a duodenal biopsy prior to initiation of a GFD.

### Survey design

The survey was developed by study team members (MS, SS) and reviewed by and modified based on input from a survey design team at the University of Michigan. The survey was then pilot tested among five physicians prior to distribution. Feedback from these providers was used to further modify the survey instrument.

The survey comprised two main sections: (1) four hypothetical patient scenarios regarding the work-up of patients with newly diagnosed IDA; and, (2) questions specifically related to the work-up and management of CD. Hypothetical patient scenarios included a 21-year-old Caucasian man, a 29-year-old African American man, a 31-year-old Caucasian woman, and a 77-year-old Caucasian man. These scenarios were selected to maximize variation on age, gender, and race. For each respondent, the first scenario presented was that of the 21-year-old Caucasian man (an individual who is at high risk for CD as a cause for his IDA due to young age, Caucasian race, and low likelihood of alternative diagnosis). The order of subsequent scenarios was randomized to minimize bias related to the subject becoming familiar with the format of the scenarios (which were identical with the exception of age, gender, and race). The first scenario read as follows: “A 21-year-old Caucasian man comes to see you complaining of generalized fatigue with no other associated symptoms. Labs show a hemoglobin of 11.1 g/dL with a mean corpuscular volume (MCV) of 72.3 fL. Iron studies reveal new iron deficiency anemia: iron level 6 ug/dL, ferritin 8 ng/mL, transferrin saturation 2%. He denies any overt bleeding. Currently, he is back for a follow up visit to discuss his results.”

In each scenario, surveyed physicians were asked which tests they would order or perform at an initial clinic visit to further work-up IDA. Options included urinalysis, iron supplementation with plans to repeat iron studies in several months, fecal occult blood testing, serologic studies for CD, referral for “open access” colonoscopy (referring directly without seeing a gastroenterologist first), referral for “open access” esophagogastroduodenoscopy (EGD), referral to hematology, or referral to gastroenterology. Participants were instructed to select all options that might apply.

The second section consisted of questions related to the diagnosis and management of CD. For instance, respondents were questioned about additional work-up in a patient with positive serologic testing for CD. Other questions asked about the approach to prescription of a gluten free diet (GFD) and referral patterns for further management of newly diagnosed CD. Data were also collected on respondent demographics, including: (1) age, gender, and race; (2) medical school affiliation; (3) years in clinical practice; (4) board certification status; and, (5) practice characteristics.

### Study population

The finalized survey was distributed electronically via the American College of Physicians (ACP) Research Center’s Internal Medicine Research panel. This panel is a representative group of ACP members who have voluntarily agreed to participate in periodic physician surveys. Participants receive points for completing surveys that may then be redeemed for gift cards. All panel members were initially invited to complete the survey. Screening questions were then used to exclude the following provider groups from the survey: (1) physician trainees; (2) retired physicians; (3) geriatricians; (4) hospitalists; and (5) physicians who spend less than 25% of their time in clinical practice. Four reminder emails to complete the survey were sent during the survey period of approximately three weeks. For this survey, respondents received 100 points (corresponding to $10).

### Statistical analysis

Survey responses were summarized using simple proportions. Multivariable logistic regression was used to identify independent predictors of testing for celiac disease. Odds ratios (ORs) and confidence intervals (CI) were calculated. Statistical significance was defined as a two-sided p-value of <0.05 for all tests. Analyses were performed using SPSS (version 21, IBM Corp., Armonk, NY) and Stata 13 (State Corp, College Station, TX).

### Institutional review board

This study was deemed to meet the criteria for exemption by the University of Michigan Institutional Review Board (IRB).

## Results

### Participant demographics

240 of 470 physicians completed the survey (51% response rate). The majority of respondents were men (62%) and Caucasian (74%) (Table 1). More than half (56%) practiced in a private office. Almost all (87%) spent more than half their time delivering primary care. Approximately half spent all of their clinical time in the outpatient setting (51%). The majority had been in clinical practice for over 20 years (52%), and most were not affiliated with a medical school (60%). Almost all respondents were board certified in Internal Medicine (98%). Additional characteristics of the study population are described in Table 1.

**Table 1:** Respondent demographics.

Physician Factor	Respondents No (%); N = 240
**Gender**	
Male	148 (61.7)
Female	92 (38.3)
**Race**	
Caucasian	178 (74.2)
African American	5 (2.08)
Asian	44 (18.3)
Native Hawaiian or Pacific Islander	0 (0.00)
American Indian or Alaskan Native	0 (0.00)
Other	13 (5.42)
**Board Certification in Internal Medicine**	
Yes	235 (97.9)
No	5 (2.08)
**Instructor or Other Faculty at a Medical School**	
Yes	97 (40.4)
No	143 (59.6)
**Patient Care Setting**	
All inpatient	3 (1.19)
Primarily inpatient with some outpatient	12 (4.74)
Primarily outpatient with some inpatient	100 (39.5)
All outpatient	129 (51.0)
Equal outpatient and inpatient	9 (3.56)
**Practice Setting**	
Private Office	134 (55.8)
University-affiliated hospital	28 (11.7)
Community hospital	26 (10.8)
Managed Care Organization	14 (5.83)
Veterans Association	15 (6.25)
Other	23 (9.58)
**Region of Practice**	
Rural	35 (14.6)
Suburban	124 (51.7)
Urban	81 (33.8)
**No. Years in Clinical Practice**	
<5 years	23 (9.58)
5–10 years	32 (13.3)
11–15 years	25 (10.4)
16–20 years	35 (14.6)
>20 years	125 (52.1)
**Percentage of Time Spent in Primary Care**	
None	3 (1.15%)
Less than 25%	6 (2.29%)
25–50%	25 (9.54%)
50% or more	228 (87.0%)
**Use of Open-Access Endoscopy**	
Yes	130 (54.2)
No	110 (45.8)

### Initial approach to iron deficiency anemia and testing for celiac disease

The approach to IDA varied widely across scenarios (p<0.001, Table 2). For example, 70% of respondents would use iron supplementation for several months in a premenopausal Caucasian woman, while fewer than 40% would use a similar approach in a male patient. 50% would refer an elderly man for open access colonoscopy, while fewer (6–12%) would refer a young patient. Notably, few would refer for open access EGD.

**Table 2:** Hypothetical patient cases of IDA.

Patient Characteristics	Iron Supplements	Referral for EGD	Referral for Colonoscopy	Serologic Testing for CD*	Referral to Gastroenterology*
21-year-old Caucasian man	86 (34.7)	28 (11.3)	26 (10.5)	151 (60.9)	53 (21.4)
77-year-old Caucasian man	74 (30.2)	54 (22.0)	123 (50.2)	44 (18.0)	95 (38.8)
29-year-old African American man	93 (38.3)	29 (11.9)	28 (11.5)	116 (47.7)	58 (23.9)
31-year-old Caucasian woman	170 (70.0)	16 (6.58)	15 (6.17)	105 (43.2)	35 (14.4)

Total number of PCPs responding in the affirmative to each survey treatment option. The number within each parenthesis indicates the percentage of total respondents.

*p<0.001 across hypothetical patient cases

**Fig 1:**
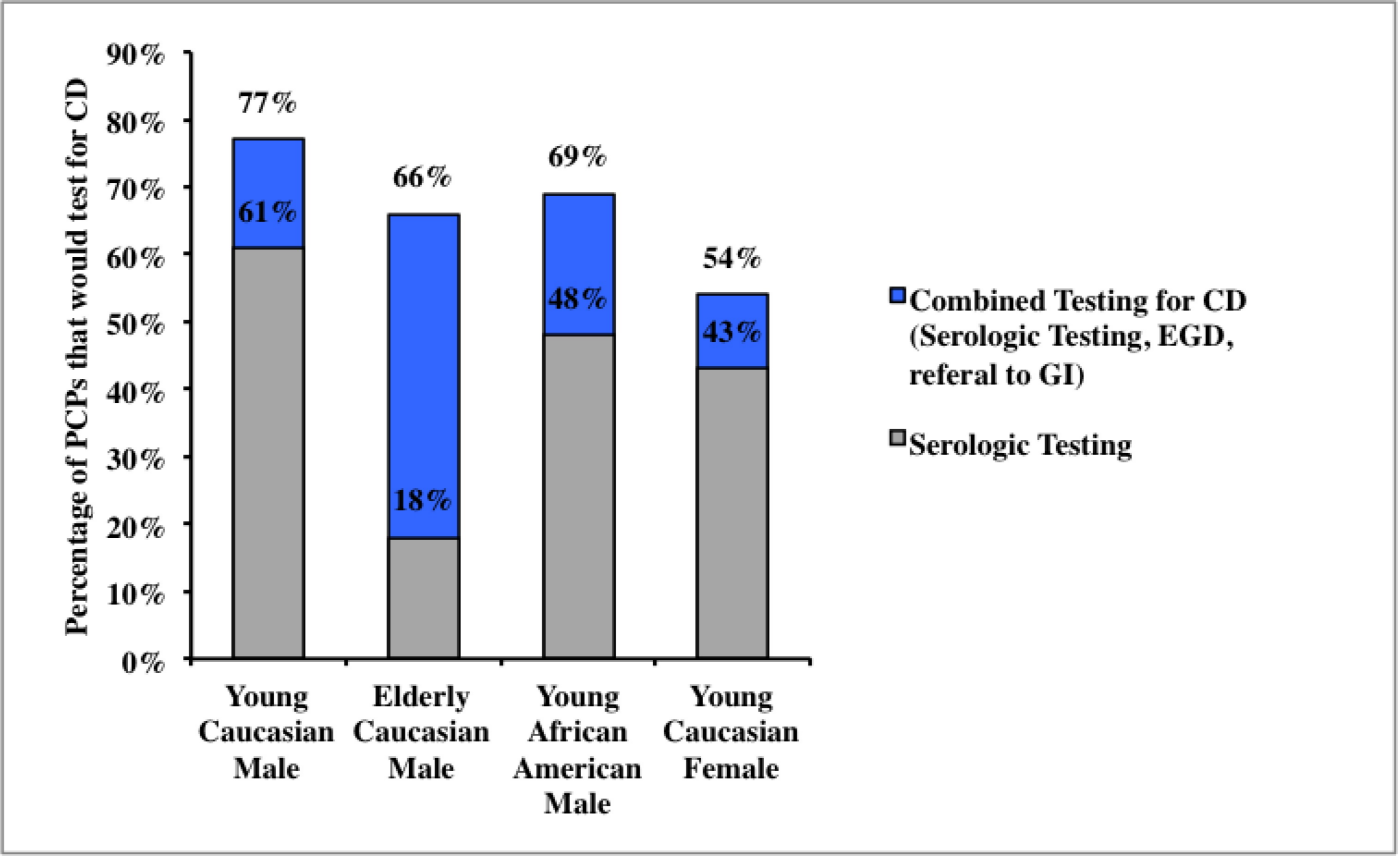
What proportion of primary care physicians test for CD in a patient with unexplained IDA?.

Frequency of testing for CD (both serologic testing and any testing, defined as serologic testing, upper endoscopy, and/or gastroenterology referral) also varied significantly according to patient characteristics. Specifically, 61% of those surveyed would perform serologic testing for CD in a young Caucasian man with IDA (77% would perform any testing), but only 18% would send for serologic testing in an elderly Caucasian man (66% would perform “any” testing). In addition, 43% of physicians would perform serologic testing for CD in a premenopausal Caucasian woman and 48% in a young African American man (the rates for any testing in these groups were 54% and 69%, respectively) (Fig 1).

In multivariable analysis, PCPs who were affiliated with an academic institution were more likely to send for serologic testing in young Caucasian men with IDA (the demographic with the highest pretest probability for CD) than PCPs who were not associated with an academic institution (OR: 2.0, 95% CI: 1.13–3.69, p-value: 0.02) (Table 3). PCPs who had been in clinical practice for 10 years or less were less likely to perform any testing for a young Caucasian man with IDA than were PCPs who were in practice for more than 10 years (OR: 0.4, 95% CI: 0.16–0.82, p-value: 0.01) (Table 4). No statistically significant association was found among a PCP’s gender, race, practice setting, years in clinical practice, or the availability of open access endoscopy and the frequency of serologic testing for CD. Similarly, a PCP’s gender, race, academic affiliation, practice setting, and availability of open access endoscopy did not predict the frequency of any testing for CD in patients with IDA.

**Table 3:** What physician factors predict serologic testing for CD in a young, Caucasian male?

	Unadjusted			Adjusted		
Physician Factor	Odds Ratio	95% CI	p value	Odds Ratio	95% CI	p value
Length in clinic practice						
>20 years	1			1		
16–20 years	1.5	0.68–3.34	0.32	1.5	0.65–3.42	0.35
11–15 years	1.8	0.69–4.55	0.23	1.5	0.56–3.97	0.43
≤ 10 years	0.9	0.47–1.69	0.72	0.7	0.32–1.34	0.25
Gender						
Female	1			1		
Male	0.9	0.52–1.51	0.65	0.8	0.47–1.49	0.55
Race						
Asian	1			1		
African American	0.7	0.11–4.67	0.71	0.6	0.09–4.59	0.65
Other	0.5	0.15–1.92	0.35	0.5	0.14–1.94	0.33
Caucasian	0.7	0.35–1.42	0.33	0.6	0.28–1.25	0.17
Academic Affiliation						
No	1			1		
Yes	1.9	1.10–3.28	0.02*	2.0	1.13–3.69	0.02*
Open Access Available						
No	1			1		
Yes	1.5	0.87–2.47	0.15	1.5	0.88–2.60	0.14
Practice Setting						
Rural	1			1		
Suburban	1.1	0.49–2.27	0.89	0.9	0.43–2.09	0.90
Urban	1.1	0.48–2.42	0.86	0.8	0.33–1.83	0.56

*Denotes Statistical Significance

**Table 4:** What factors predict “any” testing for CD (serology +/- referral for EGD +/- referral to GI) in a young, Caucasian male?

	Unadjusted			Adjusted		
Physician Factor	Odds Ratio	95% CI	p value	Odds Ratio	95% CI	p value
Length in clinic practice						
>20 years	1			1		
16–20 years	1.00	0.39–2.55	1.00	0.9	0.35–2.39	0.85
11–15 years	1.31	0.41–4.17	0.65	1.1	0.33–3.60	0.89
≤ 10 years	0.51	0.25–1.05	0.07	0.4	0.16–0.82	0.01*
Gender				** **	** **	** **
Female	1			1		
Male	0.93	0.50–1.74	0.82	0.8	0.39–1.54	0.48
Race						
Asian	1			1		
African American	0.28	0.04–2.02	0.21	0.2	0.03–1.91	0.18
Other	0.63	0.14–2.89	0.55	0.5	0.10–2.37	0.37
Caucasian	0.61	0.25–1.48	0.27	0.5	0.17–1.15	0.09
Academic Affiliation						
No				1		
Yes	1.48	0.78–2.79	0.23	1.6	0.79–3.12	0.19
Open Access Available						
No				1		
Yes	1.13	0.62–2.07	0.70	1.3	0.66–2.36	0.5
Practice Setting						
Rural	1			1		
Suburban	1.44	0.62–3.36	0.40	1.4	0.60–3.39	0.42
Urban	1.51	0.61–3.73	0.38	1.3	0.49–3.41	0.61

### Initial management of celiac disease

The majority of respondents (80%) would definitely (37%) or probably (43%) start a patient with positive serologic testing for CD on a GFD prior to confirmatory EGD. Only 13% “probably would not” and only 7% “definitely would not” start a GFD immediately after a positive serologic test (Table 5). The proportion of physicians who would send patients with a positive serology to “open access” EGD was variable: 23% “definitely would,” 30% “probably would,” 39% “probably would not,” and 7% “definitely would not”. A majority of PCPs would consider referring patients with positive CD serology to a gastroenterologist (34% “definitively would” and 38% “probably would”) (Table 5). In addition, 65% of respondents would not consider serologic testing in an elderly person with IDA, and 28% would not consider serologic testing in a premenopausal woman.

**Table 5:** Questions pertaining to positive CD serology in IDA work-up.

	Definitely Would	Probably Would	Probably Would Not	Definitely Would Not
**Immediately Start Gluten Free Diet**	90 (37.5)	103 (42.9)	31 (12.9)	16 (6.67)
**Open Access EGD**	56 (23.3)	73 (30.4)	94 (39.2)	17 (7.08)
**Referral to Gastroenterologist**	82 (34.2)	90 (37.5)	61 (25.4)	7 (2.92)

Total number of PCPs responding in the affirmative to each survey treatment option. The number within each parenthesis indicates the percentage of total respondents.

## Discussion

IDA is a common finding in clinical medicine and is the most common extra-intestinal manifestation of CD. However, data on the work-up of IDA in the primary care setting is limited. We found that testing for CD varies widely according to patient demographics, but overall, PCPs appear to underuse testing for CD in patients with IDA, regardless of the definition of testing used. In the clinical scenarios described in our survey (young Caucasian man, elderly Caucasian man, young African American man, and young Caucasian woman), PCPs only sent serologic testing in 18–61% of patients. Perhaps appropriately, elderly Caucasian men were the least likely to have serologic testing for CD performed (18%), while young Caucasian men were the most likely to undergo serologic testing. However, even for the highest risk group (young Caucasian men), only 61% of PCPs would obtain serologic testing for CD during the initial evaluation of IDA. For young Caucasian women and young African American men, less than half would send serologic testing. The proportion of PCPs who would test for CD in unexplained IDA only modestly improved when considering the more inclusive definition of any testing for CD (54–77%).

The majority of PCPs in our study would also immediately start their patients on a GFD after positive serologic testing (38% definitely would and 42% probably would). Although adhering to a GFD is important in treating and preventing complications from CD, it should not be initiated prior to endoscopic evaluation, as serology alone is insufficient to confirm the disorder [19,20]. While a positive serologic test is suggestive of the diagnosis, sensitivity and specificity of testing are variable across different laboratories (ranging from 63–93% and 96–100% respectfully) [19–21]. Moreover, small intestinal biopsies should be performed while patients are on a gluten-*containing* diet, as abstaining from gluten reduces the sensitivity of histology [19,22,23]. The results from our study should therefore raise concern that a significant proportion of patients with positive CD serology are not undergoing the appropriate confirmatory testing.

To the best of our knowledge, this study is the first of its kind to assess PCPs’ awareness of CD as a potential cause of IDA, as well as determine how diagnostic evaluation is pursued in this setting. A recent study published by Smukalla, et al, surveyed practicing hematologists to assess how often they consider CD as a cause of IDA and order the appropriate serologic testing. However, this differs from our current work, as PCPs (who see the vast majority of patients with IDA) were not included in this study [24]. In accordance with our findings, Smukalla et al found that hematologists do not routinely screen patients with IDA for CD, regardless of specific patient factors such as age, gender, race, or postmenopausal status. Contrary to our findings that PCPs with fewer years in clinical practice were less likely to screen for CD, the authors of this particular study found that hematologists who recently completed fellowship training were more likely to screen for CD. These findings were postulated to be secondary to the recent increase in recognition of CD [24]. Overall, the conclusions that hematologists under-recognize CD as a potential cause of IDA parallel our results from the primary care setting.

There were several limitations to our study. First, the sample size was relatively small, limiting our ability to draw statistical inferences. Additionally, the response rate was modest at 51%, which had the potential to introduce nonresponse bias. However, given that we surveyed a panel of physicians specifically designed to be representative of ACP members, we feel that our data fairly represent the practices of high-performing PCPs. Another limitation is difficulty in assessing whether physicians’ responses to a survey truly depict their actions in clinical practice. Future studies could examine actual use of testing for CD as opposed to self-report, though rates are likely to be lower than those reported in our study due to lack of completion of ordered testing.

In conclusion, PCPs are under-testing for CD in patients with IDA, regardless of age, gender, race, or postmenopausal status. In addition, the majority of physicians may not be strictly adhering to established guidelines regarding the diagnosis and management of CD, including confirmation of positive serologic testing with a duodenal biopsy while on a gluten-containing diet. Efforts to better educate PCPs on the importance of testing and work-up of IDA and CD are warranted.
